# Perceptions of mental health professionals towards artificial intelligence in mental healthcare: a cross-sectional study

**DOI:** 10.3389/fpsyt.2025.1601456

**Published:** 2025-07-10

**Authors:** Loujain Sharif, Reem Almabadi, Alhanouf Alahmari, Fai Alqurashi, Fidaa Alsahafi, Shahad Qusti, Walaa Akash, Alaa Mahsoon, Dev Bandhu Poudel, Khalid Sharif, Rebecca Wright

**Affiliations:** ^1^ Psychiatric and Mental Health Nursing Department, Faculty of Nursing, King Abdulaziz University, Jeddah, Saudi Arabia; ^2^ Faculty of Nursing, King Abdulaziz University, Jeddah, Saudi Arabia; ^3^ Nursing Administration Department, Eradah Mental Health Complex (Eradah Service), Jeddah, Saudi Arabia; ^4^ Nursing Department, Eradah Mental Health Complex, Jeddah, Saudi Arabia; ^5^ Department of Humanities and Social Sciences, G.P. Koirala Memorial (Community) College, Kathmandu, Nepal; ^6^ Department of Behavioral Medicine and Psychiatry, West Virginia University, Morgantown, WV, United States; ^7^ School of Nursing, Johns Hopkins University, Baltimore, MD, United States

**Keywords:** artificial intelligence, mental healthcare, perceptions, psychiatry, Saudi Arabia

## Abstract

**Background:**

Artificial intelligence (AI) holds significant potential for enhancing mental health care, but uptake is limited, potentially impacted by demographic factors of healthcare professionals. Further, while AI use in Saudi Arabia is progressive, there is minimal exploration of its role and impact within mental health services.

**Objective:**

This study presents a unique exploration of psychiatric professional’s perceptions of AI in mental health care in Jeddah, Saudi Arabia.

**Methods:**

A cross-sectional online survey was conducted with a sample of mental health professionals from two governmental mental health hospitals in Jeddah, Saudi Arabia. The study tool was made up of two sections, the first consisting of sociodemographic questions and the second was the Shinners Artificial Intelligence Perception (SHAIP) questionnaire assessing the perceptions towards AI in mental healthcare, with data analyzed using IBM SPSS Statistical software.

**Results:**

A total of 251 mental health professionals, mostly females (56.6%), aged 31-40 (50%), married (45%), and nurses (55.4%). Only 24.3% used AI in practice, though 85.7% were aware of AI. Participants positively rated AI’s impact (mean item range: 3.48-3.75) and felt unprepared for role-specific AI (mean 2.78). Nurses and those aware of AI had higher AI impact perceptions (p<0.0001) Specialty and AI awareness affected AI preparedness (p=0.001, p=0.029).

**Discussion:**

The study provides insights into mental health professionals ‘ views on AI in mental healthcare, emphasizing the need for targeted education to improve AI literacy and preparedness among Saudi healthcare professionals. It highlights the importance of ethical AI implementation to enhance patient care and advance psychiatric practice in the region.

## Introduction

The World Health Organization (WHO) describes mental health as a state of well-being where an individual recognizes their abilities, manages everyday stress, works effectively, and contributes to their community (1). Mental health disorders include various issues such as depression, anxiety, addiction, bipolar disorder, and other disorders, which significantly affect a person’s daily life, relationships, and physical health (2). In recent years there has been rapid uptake and integration of Artificial Intelligence (AI) in mental healthcare with the goal of improving patient care through greater efficiency in processes, prediction, and resources to individualize care (3, 4). While there is no standard definition for AI, the American Psychological Association defines it as ‘a subdiscipline of computer science that aims to produce programs that simulate human intelligence’ (5).

In practice there are different types of AI including machine learning – neural network and deep learning; and natural language processing (NLP); rule-based expert systems; robotic process automation and physical robots (6, 7). Research in artificial intelligence (AI) has primarily focused on components such as learning, reasoning, problem-solving, decision making, creativity, perception, independence and language use (8–10). Benefits of AI include improved efficiency, accuracy, and productivity (11). AI has been extensively used in health care system (12), including mental health systems (3, 13, 14). (6, 15–21) Attitudes toward AI in healthcare are often positive (22–24), with professionals noting the potential of AI to support medical diagnosis, decision-making, drug discovery, patient experience, data management, and robotic surgery (25, 26). However, an important distinction is the perspective among many healthcare professionals is that AI should serve as a partner not a replacement as a way of maximizing the benefits of AI and the expertise of the clinician (27). Other factors raised by healthcare professionals reflecting on the integration of into healthcare relate to job security, ethical and moral dilemmas, impact on quality of care including patient-provider relationships, training needs, and legal implications and regulatory standards (27). AI-driven advancements can improve personalized, precise, and predictive care, while challenges to integration include data access, domain expertise, public trust, ethical concerns, cybersecurity, and language barriers (12, 13, 22). Additionally, effective AI implementation depends on robust evaluation, bias mitigation, generalizability, and interpretability to maximize patient benefits (28). However, limited digital literacy remains a barrier, with studies showing that nearly half (48.2%) of healthcare professionals exhibit poor digital literacy, which may limit use of AI in supporting clinical practice (29).

### AI in mental health

Advances in AI offer promising opportunities for patients and clinicians alike, to benefit from mental health interventions (3, 5, 13). AI tools have demonstrated accuracy in detecting, classifying, and predicting mental health disorders, assessing treatment responses, and monitoring prognosis (14). Additionally, AI-driven mental health interventions, including digital apps and web-based therapy, are improving patient access and individualized care (18). AI-based mental healthcare systems (AIMS) integrate chatbots with machine learning to assess patients, predict conditions, and support clinical decisions while prioritizing privacy, autonomy, and accessibility (19). Further, impact on patient outcomes via chatbot-based systems show potential in psychoeducation, treatment adherence, and patient engagement by analyzing energy levels, mood, stress, and sleep habits (4, 15). In addition, in some instances patients have responded positively to the care not coming from a human, based on the control this allowed them in self-pacing treatments and education (30). Machine learning models can predict conditions such as bipolar disorder, schizophrenia, anxiety, depression, post-traumatic stress disorder (PTSD), and childhood mental health disorders (20). Though AI can analyze speech, text, and facial expressions to provide insights into mental states (6) and detect patterns related to non-compliance (31), encoding human empathy into AI remains a fundamental challenge (16).

Thus, while preliminary evidence supports the use of chatbots in psychiatry (21), effectiveness requires technological advancement, resource availability, and reduction of stigma (16), ethical concerns remain. Ethical implications are wide-reaching, including but not limited to potential harm to patients, biases and lack of representation in datasets, over-reliance, limited emotional intelligence, and challenges in preserving the patient-therapist relationship (15, 30, 32). A recent systematic review identified 18 ethical considerations within three main domains: use of AI interventions in mental health and wellbeing, principles to ensure responsible practice and positive outcomes associated with development and implementation of AI technology, and guidelines and recommendation for ethical use of AI in mental health treatments (32). To be trustworthy, AI-driven mental healthcare must be competent, reliable, transparent, and empathetic, along with clear communication about its validity and limitations (17). Despite AI’s rapid evolution in healthcare, its application in mental health is also limited by barriers in clinical integration (28, 33). Digital literacy among clinicians is becoming increasingly essential as AI-generated data grows (34). AI presents transformative potential, but ensuring ethical implementation and clinical effectiveness will be key to its success in mental healthcare (3).

### Use of AI in healthcare in Saudi Arabia

AI is rapidly transforming healthcare in Saudi Arabia, aligning with Vision 2030 by enhancing efficiency through AI-powered diagnostics, predictive analytics, personalized treatments, and digital platforms like the Mawid system, which streamlines scheduling and service delivery (35). These advancements contribute to improved patient outcomes and operational effectiveness across the healthcare sector. Despite AI’s growing role in Saudi, knowledge gaps persist among its healthcare professionals. Studies indicate that 50.1% of medical students lack basic AI knowledge, 55.8% are unaware of its applications in dentistry, and 40.9% primarily learn about AI through social media. AI awareness increases with academic progression, with first-year students showing the lowest awareness (27.6%) and sixth-year students the highest (64.6%) (36), which provides some explanation for greater support for AI education among postgraduate (48.9%) than undergraduate (40.4%) programs (36). In the field of mental health, AI is emerging as a valuable tool for diagnosis, treatment, and accessibility. King Faisal Specialist Hospital & Research Centre (KFSHRC) is leveraging AI-driven digital platforms to enhance mental health services, reduce stigma, and improve care access, particularly in underserved areas (37). These initiatives highlight AI’s potential to bridge gaps in mental healthcare, but further efforts are needed to integrate AI effectively, ensuring healthcare professionals are equipped with the necessary digital skills.

### Research gap

AI perceptions among mental health professionals appear to be influenced by demographic factors though exploration shows inconclusive and contradictory findings. For example, age plays a role, with younger professionals generally more receptive, though findings are mixed (38–40). (24, 41) Gender differences are also inconclusive, with some studies suggesting men favor AI due to socialization (42), while others report higher acceptance among women (24). Specialization also shapes attitudes, with pathologists showing greater willingness compared to other medical specialists, such as psychiatrists, radiologists, and surgical specialists (39), and cognitive behavioral practitioners demonstrating more positive views (43). Exploration of experience, digital literacy and AI familiarity also give mixed findings, wherein experience is not a strong predictor, and digital literacy and AI familiarity being found to both enhance or diminish positive perceptions (40, 42, 44). (39) Understanding the interplay of these demographic influences is essential for effective AI adoption in mental healthcare.

Despite the exploration highlighted, literature on the intersection of AI and mental health remains relatively scarce (45). AI adoption in healthcare is expanding, but research on its role in mental health care in Saudi Arabia remains limited. Existing studies primarily focus on general healthcare applications, overlooking demographic variations in AI perception, particularly in mental health care. There is a lack of research examining how factors such as age, gender, professional background and experience influence attitudes toward AI in mental health settings. Thus, there is a need to identify insights for developing targeted strategies to enhance AI integration, improve training programs, and optimize AI tool usage, ultimately fostering more effective and accessible mental health care in Saudi Arabia. Therefore, this study addresses this gap with an aim of exploring AI perceptions in mental health care across diverse demographic groups in Saudi Arabia.

## Methods

### Research design

A cross-sectional study was conducted to assess the perceptions of mental health professionals towards AI technology in mental healthcare. The study focused on two specialized psychiatric hospitals in Jeddah, Saudi Arabia: XX and XX. These institutions, affiliated with the Ministry of Health (MOH), were selected due to their specialization in psychiatric care and location. XX, established in 1988, provides comprehensive psychiatric care, while XX features eight wards, including six inpatient units (four male and two female wards), an outpatient department (OPD), and an emergency room (ER), totaling approximately 125 beds.

### Ethical considerations

Ethical approval was obtained from the Nursing Research Ethical Committee of The Faculty of Nursing at King Abdulaziz University (NREC Serial No: Ref No 2B. 45) and the Institutional Review Board of The Ministry of Health (IRB Log No: A01892). Participants were informed about the study’s aim, importance, expected completion time, and confidentiality measures through a cover page included in the online survey. Informed consent was implied upon survey completion. Data anonymity was ensured by not collecting personal identifiers, and data security was maintained using password-encrypted storage accessible only to the research team.

### Sampling and sample size

The study aimed to include a diverse range of Mental Health Professionals that are representative of clinicians providing direct care to individuals with mental health disorders in Saudi Arabia. The sample therefore included psychiatrists, medical interns, nurses, nursing interns, psychologists, psychologist interns, sociologists, and other allied health professionals. Assuming 60% of study subjects are having good knowledge towards AI with ± 6% precision and at 0.05 level of significance we need 253 subjects. These 253 subjects were selected using systematic random sampling from the sampling frame of 705 professionals across both hospitals.

### Instrumentation/data collection method

Data were collected via a questionnaire comprised of two sections and a total of 19 questions. Section one comprised the validated Shinners Artificial Intelligence Perception (SHAIP) questionnaire (used with author permission) (46). The SHAIP tool was selected for use in this study due to the methodological rigor and strength in psychometric validation (46). The SHAIP questionnaire consists of 10 items assessed using a 5-point Likert scale (totally disagree, disagree, unsure, agree and total agree), exploring perceptions of professional impact (Cronbach’s alpha.804), and perceptions of preparedness for AI (Cronbach’s alpha.620) (46). Within the ten items, six indicate professional impact: I believe that the use of AI in my specialty could improve the delivery of patient care; I believe that the use of AI in my specialty could improve clinical decision making; I believe that AI can improve population health outcomes; I believe that AI will change my role as a healthcare professional in the future; I believe that the introduction of AI will reduce financial cost associated with my role; I believe that one day AI may take over part of my role as a healthcare professional. Four items explore preparedness for AI: I believe that overall healthcare professionals are prepared for the introduction of AI technology; I believe that I have been adequately trained to use AI that is specific to my role; I believe there is an ethical framework in place for the use of AI technology in my workplace; I believe that should AI technology make an error; full responsibility lies with the healthcare professional. The second section comprised nine socio-demographic questions reflecting the same information gathered by Shinner et al. (46). These included: age, gender, marital status, role including (i) professional role, and (ii) clinical or administrative role, years of experience in mental health care, and AI use including (i) current use of AI in practice, (ii) any prior AI training, and (iii) ability to define AI. The questionnaire takes on average 5–10 minutes to complete.

### Data collection procedure

A standardized approach to data collection was employed to facilitate robust statistical analysis and derive meaningful insights into professionals’ perceptions of AI integration in mental healthcare. The questionnaire included language about the voluntary nature of participation, anonymization of responses, and a statement that completion of the questionnaire indicated informed consent. Following ethical approval, potential participants were notified about the study using electronic distribution via hospital emails and official WhatsApp groups to ensure participant anonymity and accessibility. The questionnaire remained active for a one-month period between April-May 2024, and two electronic reminders were sent to encourage completion. The survey was closed once the target sample size had been achieved.

### Data analysis

Data were analyzed using IBM SPSS Statistical software for Windows version 26.0 (IBM Corp., Armonk, N.Y., USA). Descriptive statistics (mean, standard deviation, frequencies and percentages) were used to describe the quantitative and categorical variables. The student’s t-test for independent samples and one-way analysis of variance followed by Tukey’s *post-hoc* tests were used to compare the mean values of two factors of SHAIP questionnaire in relation to the demographic, professional characteristics and AI awareness items of the study participants which had two and more than two categories. A p-value of <0.05 were used to report the statistical significance of results.

## Results

Out of 251 study participants, 56.6% were females, about 50% were in age group of 31 to 40 years, 45% were married. Professionally, 55.4% were nurses, and 83.1% were in a clinical role. Work experience varied between 5–10 years (27.5%), and 11–20 years (35.9%) (**Table 1**).

**Table 1:** Demographic characteristics of participants (N = 251).

Characteristic	n	%
Age (years)		
<20	17	6.8
21-30	74	29.5
31-40	125	49.8
>40	35	13.9
Gender		
Male	109	43.4
Female	142	56.6
Marital status		
Married	113	45.0
Engaged	22	8.8
Single	99	39.4
Others	17	6.8
Specialty		
Nurse	139	55.3
Nurse intern	24	9.6
Psychiatrist	20	8.0
Psychologist	34	13.5
Sociologist	16	6.4
Other	18	7.2
Field of Specialty		
Administrative	39	16.9
Clinical	192	83.1
Years of experience		
<5	79	31.5
5 to 10	69	27.5
11-20	90	35.8
>20	13	5.2

Percentages are calculated based on total sample size (N = 251).

Use or familiarity with AI varied across the sample. While the majority claimed they knew what AI was (85.7%), most had not received any training in its use (72.9%). Notably, while 66.5% stated they were not using AI in their clinical practice, and 24.3% stated they were using it, 9.2% indicated they were unsure (**Table 2**).

**Table 2:** Distribution of study subject’s responses towards Artificial Intelligence.

Items	Response	n (%)
Are you currently using Artificial Intelligence (AI) in your clinical practice?	Yes	61 (24.3)
	No	167 (66.5)
	I don’t know	23 (9.2)
Have you received any courses or training about Artificial Intelligence?	Yes	68 (27.1)
	No	183 (72.9)
Do you know what Artificial Intelligence is?	Yes	215 (85.7)
	No	36 (14.3)

Participant responses to the SHAIP questionnaire highlight important complexities within their attitudes towards AI (**Table 3**). For example, 61.8% indicated ‘agree’ to the item ‘*I believe that AI can improve population health outcomes*’, 53.8% agreed that AI would change their healthcare role, and 55% agreed AI may take over portions of their healthcare role. However, responses to preparedness items suggest contradictory perceptions among participants. Only 20.3% agreed they had been adequately trained to use AI within their specialty role, affirmed by 32.7% and 25.1% stating unsure and disagree respectively, yet 48.2 agreed that the healthcare professionals are prepared for the introduction of AI technology.

**Table 3:** Healthcare professionals’ attitudes toward AI implementation (N = 251).

Questions	Totally disagree n (%)	Disagree n (%)	Unsure n (%)	Agree n (%)	Totally Agree n (%)	Mean (SD)
I believe that the use of AI in my specialty could improve the delivery of patient care.	10 (4.0)	16 (6.4)	50 (19.9)	130 (51.8)	45 (17.9)	3.73 (0.96)
I believe that the use of AI in my specialty could improve clinical decision making.	9 (3.6)	22 (8.8)	58 (23.1)	126 (50.2)	36 (14.3)	3.63 (0.96)
I believe that AI can improve population health outcomes.	13 (5.2)	12 (4.8)	36 (14.3)	155 (61.8)	35 (13.9)	3.75 (0.94)
I believe that AI will change my role as a healthcare professional in the future.	11 (4.4)	21 (8.4)	56 (22.3)	135 (53.8)	28 (11.2)	3.59 (0.95)
I believe that the introduction of AI will reduce financial cost associated with my role.	11 (4.4)	13 (5.2)	69 (27.5)	134 (53.4)	24 (9.6)	3.59 (0.90)
I believe that overall healthcare professionals are prepared for the introduction of AI technology.	5 (2.0)	28 (11.2)	72 (28.7)	121 (48.2)	25 (10.0)	3.53 (0.89)
I believe that one day AI may take over part of my role as a healthcare professional.	19 (7.6)	25 (10.0)	46 (18.3)	138 (55.0)	23 (9.2)	3.48 (1.04)
I believe that I have been adequately trained to use AI that is specific to my role.	38 (15.1)	63 (25.1)	82 (32.7)	51 (20.3)	17 (6.8)	2.78 (1.14)
I believe there is an ethical framework in place for the use of AI technology in my workplace.	6 (2.4)	15 (6.0)	65 (25.9)	121 (48.2)	44 (17.5)	3.73 (0.90)
I believe that should AI technology make an error; full responsibility lies with the healthcare professional.	15 (6.0)	26 (10.4)	66 (26.3)	110 (43.8)	34 (13.5)	3.49 (1.04)

**Figure 1:**
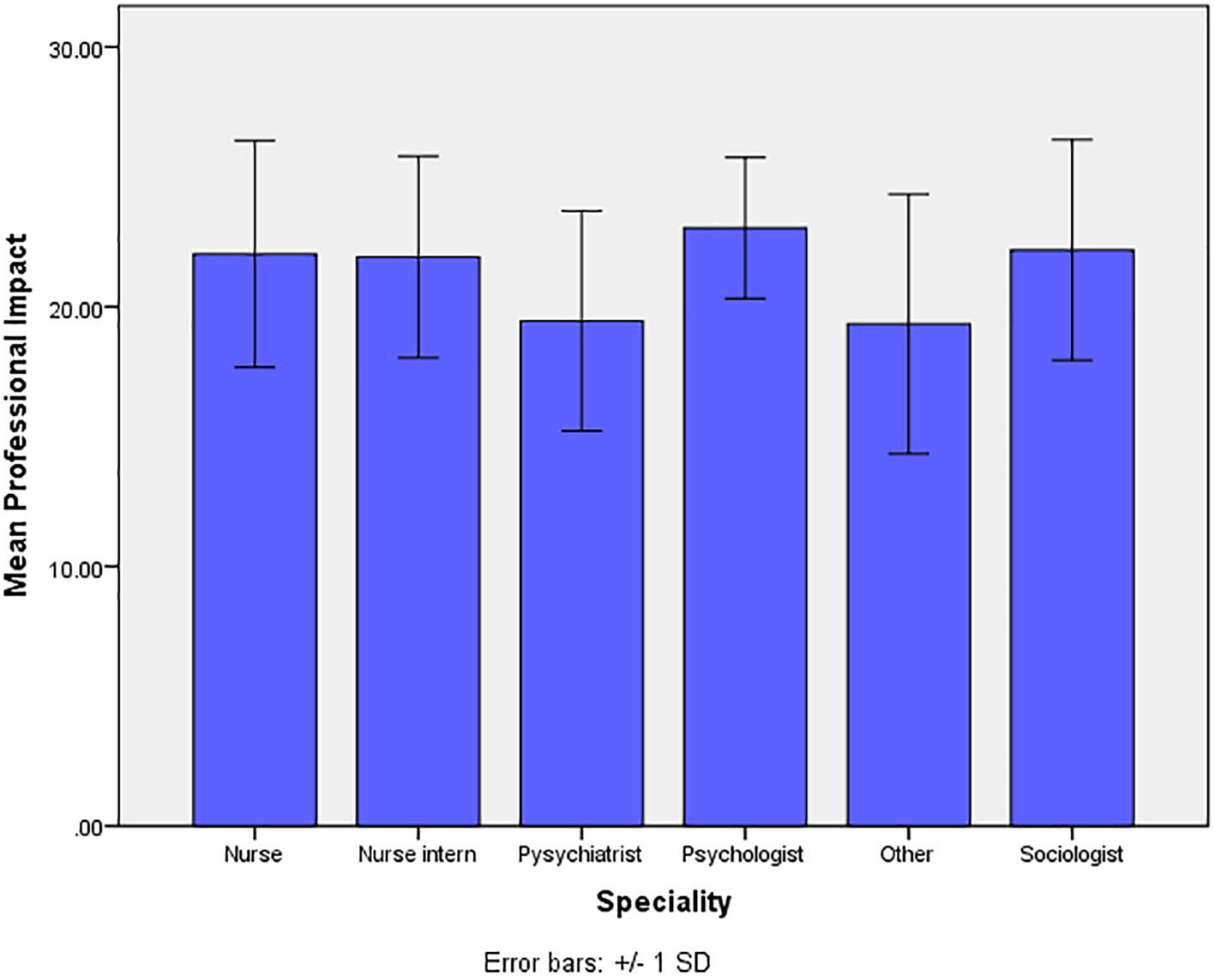
Presentation of mean values for professional impact and preparedness of AI.

The mean (Sd.), of the two factors under exploration (Professional Impact of AI, Preparedness of AI), are 21.76(4.26), and 13.52(2.88) respectively (**Figure 1**). The comparison of mean values of the two factors of the SHAIP questionnaire in relation to the demographic, professional and awareness items on AI show statistically significant differences (**Table 4**). These were found within the variable of ‘specialty’ and the personal profile characteristic ‘*do you know what AI is?’* for both factors of professional impact of AI and preparedness of AI. The variable ‘age’ however, was also found to be significant for differences found within the factor of professional impact of AI. The mean values of professional impact of AI are significantly different across the 4 age groups (p=0.003). The mean values of participants are significantly higher in those aged <20, 31–40 and > 40 years when compared with the participants who are aged 21–30 years.

**Table 4:** T-Test and ANOVA results for the SHAIP questionnaire.

Characteristic	Professional Impact M (SD)	*p-value	Preparedness M (SD)	*p-value
SHAIP Questionnaire	21.76 (4.26)		13.52 (2.88)	
Age groups (years)		0.003		0.456
<20	21.59 (4.71)		13.82 (3.22)	
21-30	20.46 (4.77)		13.45 (3.32)	
31-40	22.72 (3.71)		13.71 (2.56)	
>40	21.20 (4.08)		12.85 (2.84)	
Gender		0.109		0.871
Male	22.25 (3.73)		13.56 (2.64)	
Female	21.39 (4.60)		13.50 (3.06)	
Marital status		0.179		0.537
Married	22.08 (4.17)		13.57 (2.58)	
Engaged	22.18 (3.86)		13.14 (2.96)	
Single	21.65 (4.32)		13.70 (3.00)	
Others	19.70 (4.66)		12.70 (3.93)	
Specialty		0.008		0.001
Nurse	22.03 (4.36)		13.93 (2.80)	
Nurse intern	21.92 (3.87)		13.62 (2.33)	
Psychiatrist	19.45 (4.23)		10.95 (2.93)	
Psychologist	23.03 (2.71)		13.88 (2.21)	
Sociologist	22.18 (4.24)		12.62 (3.64)	
Other	19.33 (4.99)		13.28 (3.15)	
Field of Specialty		0.096		0.441
Administrative	20.85 (4.93)		13.23 (3.24)	
Clinical	22.07 (4.0)		13.61 (2.74)	
Years of experience (years)		0.321		0.507
<5	21.67 (4.53)		13.86 (3.08)	
5 to 10	22.16 (3.75)		13.40 (2.72)	
11-20	21.83 (4.34)		13.44 (2.76)	
>20	19.76 (4.43)		12.69 (3.35)	
Are you currently using Artificial Intelligence (AI) in your clinical practice?		0.058		0.778
Yes	21.11 (5.91)		13.40 (3.72)	
No	22.19 (3.54)		13.61 (2.55)	
I don’t know	20.34 (3.37)		13.21 (2.69)	
Have you received any courses or training about artificial intelligence?		0.057		0.777
Yes	20.93 (5.67)		13.44 (3.67)	
No	22.07 (3.56)		13.55 (2.54)	
Do you know what Artificial Intelligence?		<0.0001		0.029
Yes	22.17 (4.20)		13.69 (2.83)	
No	19.30 (3.84)		12.55 (3.06)	

*p <.05. M, Mean; SD, Standard Deviation.

The bold values represent statistically significant p-values (i.e., p < .05).

**Figure 2:**
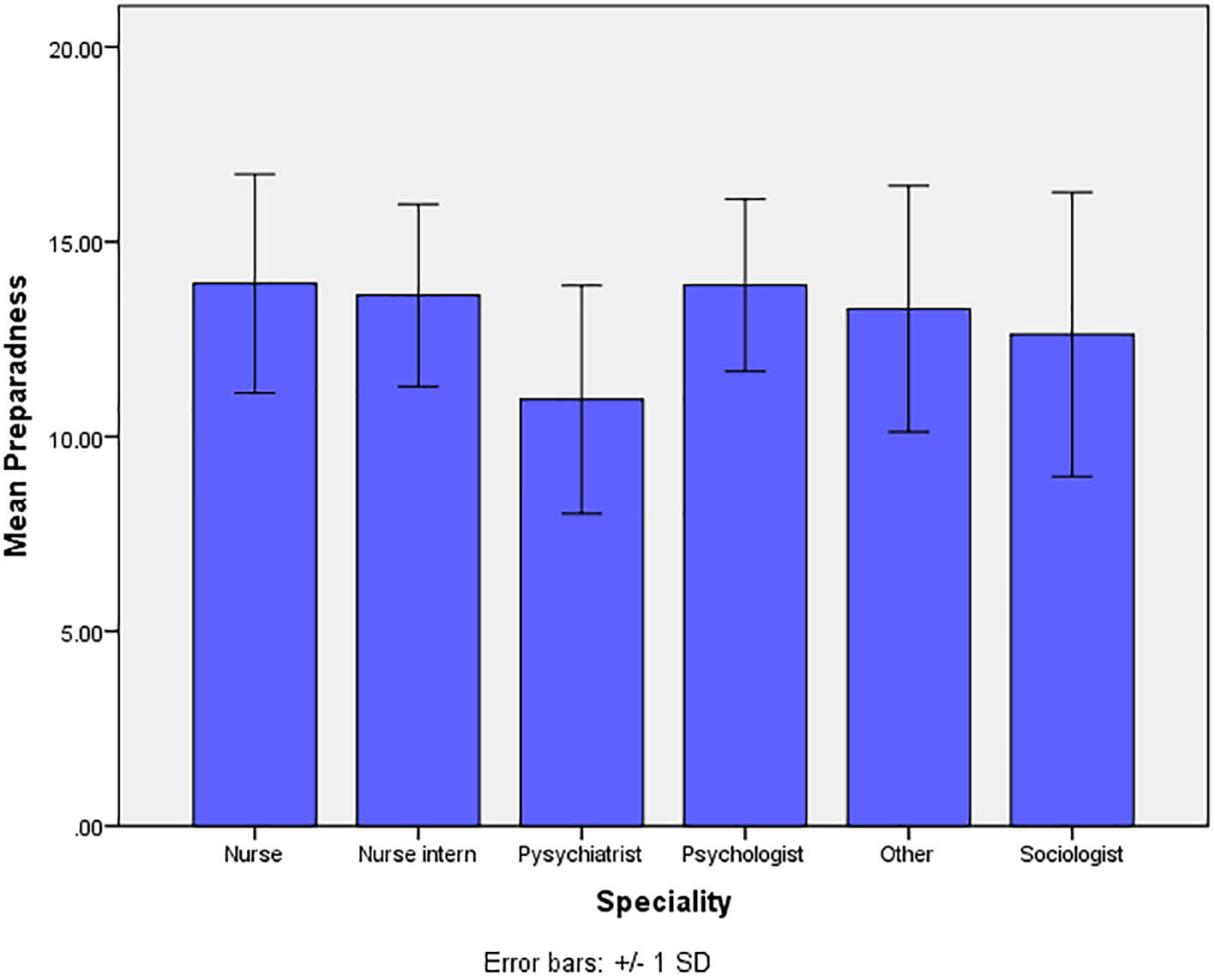
Mean values of professional impact of AI by professional specialty.

The mean values of professional impact of AI are significantly higher in participants who were nurses, nurse interns, psychologists, and sociologists when compared with the mean values of participants who were psychiatrists and of other specialties (p=0.008) (**Figure 2**). The *post-hoc* test indicates no significant difference in the mean values of pairs of nurses, nurse interns, psychologists and sociologists. The mean value of professional impact of AI is significantly higher among participants who had responded positively (yes) to the statement ‘*do you know what AI is*?’ when compared with the participants who had responded negatively (No) (p<0.0001). No statistically significant difference was observed for the mean values of professional impact of AI in relation to the other variables (**Table 4**).

**Figure 3:**
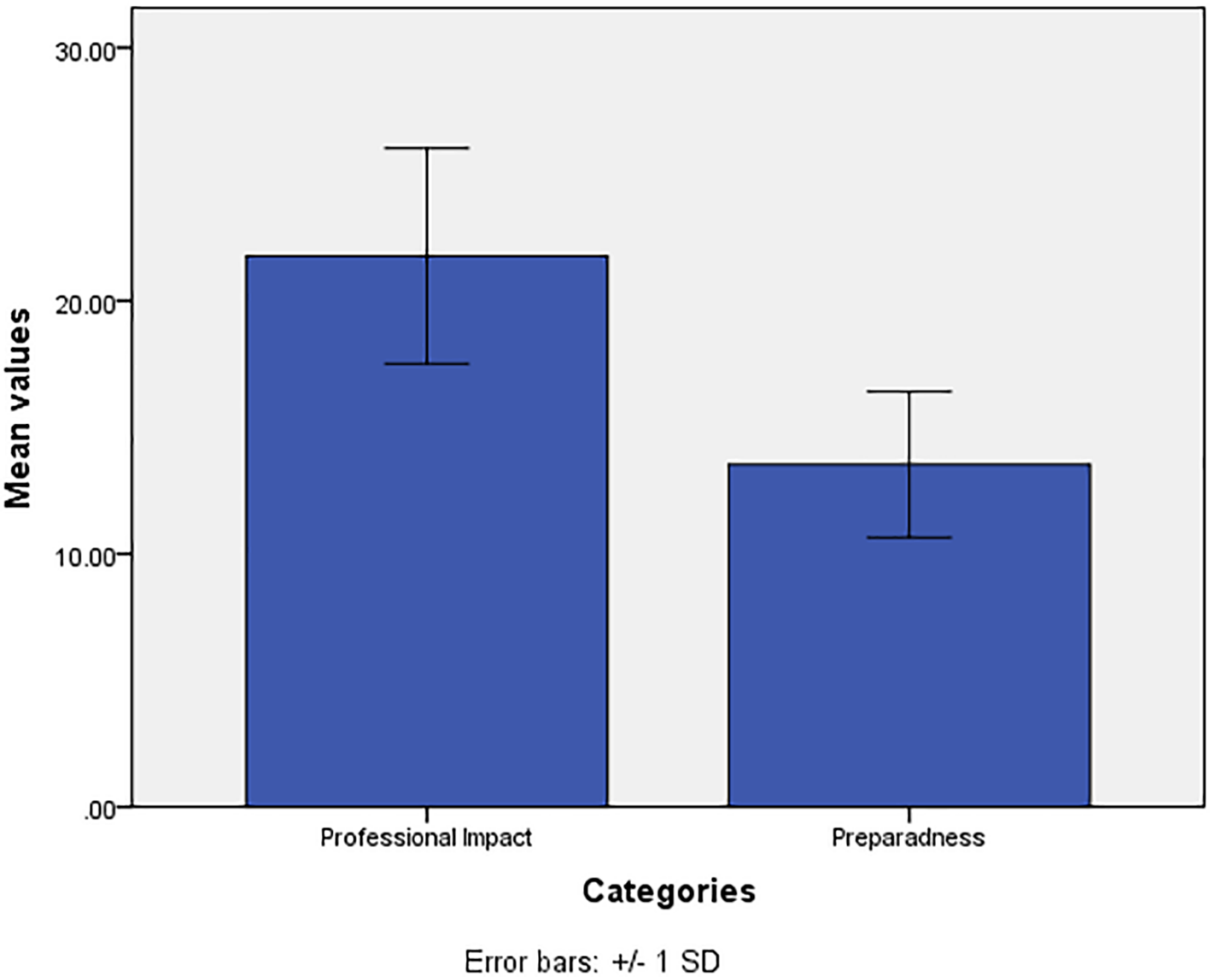
Mean values of preparedness for AI by professional specialty.

For the mean values of preparedness of AI, we observed statistically significantly higher difference in the mean values among the participants who were nurses, nurse interns, psychologists, and other specialties when compared with the mean values of participants who were psychiatrists and sociologists (p=0.001) (**Figure 3**). The *post-hoc* test indicates no significant difference in the mean values of pairs of nurses, nurse interns, psychologists and other specialties. The mean value of preparedness of AI are significantly higher among participants who had responded positively (yes) to the statement ‘*do you know what AI?*’ when compared with the participants who had responded negatively (No) (p=0.029). No statistically significant difference was observed for the mean values of preparedness of AI in relation to the other variables (**Table 4**).

## Discussion

This cross-sectional study aimed to explore perceptions of AI among a diverse range of mental healthcare professionals in Saudi Arabia. The study sample consisted predominantly of individuals aged 31–40 years (49.8%), with a significant representation of females (56.6%). A large proportion of participants were married (45.0%), compared to single (39.4%). Professionally, nurses constituted the majority (55.4%), followed by psychologists (13.5%) and nurse interns (9.6%). Most participants worked in clinical roles (83.1%), with a smaller percentage in administrative positions (16.9%). Over a third of participants had 11–20 years of clinical experience (35.9%), while a significant portion had less than 5 years (31.5%). These demographic trends highlight a sample with diverse professional backgrounds and experience levels, which may influence their perceptions and preparedness for AI integration in healthcare. Notably, the underrepresentation of certain specialties, such as psychiatrists (8.0%) and sociologists (6.4%), may limit the generalizability of findings across all healthcare professions.

The findings of this study shed light on three key elements connected to AI preparedness and perceived impact among mental health professionals in Saudi Arabia. First, we found there was an overarching general awareness of AI, but a lack of training supporting its use. This suggests a potential risk for poor or unsafe practices if AI interventions are implemented without establishment of institutional infrastructure to provide training and support. The second area pertains to the perceived professional Impact of AI. Overall, participants were largely positive their perceptions of the role of AI in mental healthcare. However, while about half believed AI would alter their clinical role and potentially take on aspects of their responsibilities, the survey did not capture if they felt this change would also be a positive move – an area requiring further investigation. The third element builds on this aspect, with notable differences in perceptions and preparedness between different professional roles and demographics (explored further below). Nurses, psychologists, and sociologists reported a greater level of perceived impact and preparedness than psychiatrists and some other professionals, associated with higher levels of knowledge. This reinforces the need for training to accompany intervention development if AI is to be integrated to greater effect in the clinical setting. It further suggests a relationship between familiarity and attitude.

Age revealed complex relationships between demographic factors and AI perception. Age emerged as a significant predictor of professional impact but not preparedness, adding nuance to existing literature. While some studies found younger age groups (18–42 years) more receptive to AI mental health tools (38), others reported no significant age-related differences in AI perception and attitudes (24, 39, 40). (41) The age-related variation in our study appears more closely tied to professional impact rather than general preparedness, suggesting that age influences how professionals view AI’s effect on their work rather than their readiness to adopt it. This is further supported by Brinker et al. (2019) who found that diagnostic performance patterns vary with age—younger clinicians showing higher sensitivity and older clinicians better specificity—indicating that AI tools may need to be tailored to different experience levels (47).

Gender showed no significant relationship with AI perception in our study, aligning with several recent studies (39, 40). However, this finding exists within a complex landscape of contradictory evidence. Ofosu-Ampong (2023) found men perceived AI-based learning tools more positively, attributing this to traditional gender roles and socialization patterns (42). Conversely, Fritsch et al. (2022) reported women showed more favorable attitudes toward AI in healthcare (24). Gender differences were also observed in diagnostic performance, with female clinicians showing higher sensitivity and male clinicians greater specificity (47). These contradictory findings suggest the presence of unmeasured moderating variables that warrant further investigation. Our study proved marital status to be a non-significant factor in AI perception, though this demographic variable has received limited attention in previous research.

Professional specialization emerged as a crucial factor in shaping AI perceptions. Psychologists reported the highest professional impact scores, while nurses demonstrated the highest preparedness levels. This finding is particularly noteworthy as previous research often excluded these key healthcare professionals (39). Within mental health specialties, those practicing cognitive behavioral approaches showed more positive attitudes toward AI (43). However, the adoption landscape remains complex, particularly in psychiatry, where evidence gaps regarding AI’s benefits and limitations make implementation decisions challenging (16). This specialty-specific variation in AI perception suggests the need for tailored implementation strategies that consider each profession’s unique needs and concerns. However, our study found no relationship between field of specialty, such as administrative and clinical roles, and AI perception. Participants from both fields exhibited no differences in AI perception across both factors. Years of experience showed no significant relationship with AI perception, consistent with previous findings (39, 40). However, some nuanced differences emerged: senior physicians were reported to be less familiar with AI (40), and more experienced doctors tended to prioritize human expertise over AI in diagnostics (44).

In our analysis, the current use of AI in mental health care settings did not reveal a relationship with AI perception in either professional impact and AI preparedness. We observed no connection between professional’s training in AI and AI perception. Interestingly, professionals who received training and professionals who had not, had similar levels of perceptions towards AI. While in the literature, digital literacy and training in technology emerged as key factors influencing healthcare professionals’ attitudes toward AI adoption (29), in our work, the relationship between professional’s knowledge and AI perception proved significant. Specifically, that those familiar with AI showed a more positive perception. This aligns with findings on technology familiarity’s importance (42), though again, wider literature is not consistent on this relationship (39).

From a cultural perspective, our findings among mental healthcare professionals are reflected among other healthcare specialties in the region. For example, a cross-sectional study in Jeddah, Saudi Arabia found healthcare workers, including mental health professionals, demonstrated substantial awareness of AI, but limited AI experience and training (48). Similarly, a study from the United Arab Emirates pointed to positive attitudes towards the role of AI in certain administrative clinical processes, with limited training and education opportunities (49). More globally, perceptions towards the potential role of AI include questions about its ability or appropriateness to replace human connection and empathy in mental healthcare, along with concerns about ethical elements (50). However, a systematic review about the future role of AI in Saudi healthcare more generally, suggests that its potential benefits in improving care processes mean the work to ensure ethical application is a worthwhile endeavor (51). Within this wider context, the findings from the present study suggest that in Saudi Arabian mental healthcare, gaps in level of preparedness can be supplemented by rigorous training across professions, a need for more work to ensure safe and ethical integration of AI to improve patient care processes, and support and partnership with mental healthcare professionals in understanding the potential evolution of their roles and responsibilities.

### Strengths and limitations

This study has several strengths and limitations. The findings of the study underscore the need for targeted training and education for mental health professionals regarding AI technologies. Furthermore, it highlights the importance of addressing concerns and misconceptions that may hinder the adoption of AI in mental healthcare. Given the mixed responses towards AI, the use of anonymous reporting may have helped to mitigate social desirability and response biases. Its cross-sectional design captures perceptions at a single point in time, limiting the ability to assess changes over time. The study was conducted in two specialized psychiatric hospitals in Jeddah, which may restrict the generalizability of findings to other psychiatric settings or healthcare sectors, though the settings were selected due to their specialized nature. Additionally, while the survey demonstrated acceptable reliability, some sections had Cronbach’s alpha values slightly above 0.5, indicating potential limitations in internal consistency. The sample relied on electronic distribution, which may have led to selection bias, favoring participants more comfortable with digital platforms. Lastly, unmeasured factors such as prior AI exposure, institutional policies, and cultural attitudes toward technology may have influenced responses, although this was beyond the scope of our primary research aim. Nonetheless, this is an important area for future researchers to consider, with use of longitudinal designs, further exploration across other psychiatric settings to improve generalizability, and qualitative exploration to provide context and further nuance to better understand mental health professionals’ attitudes, perspectives, and needs for integrating AI into patient care.

## Conclusion

Our findings indicate that there are relationships between the demographic variables of healthcare professionals and perceptions and use of AI in practice. Specialization and AI knowledge significantly influence perceptions among mental health professionals. Psychologists reported the highest professional impact, while nurses showed the greatest preparedness. Psychiatrists and sociologists exhibited lower preparedness scores, highlighting potential gaps. Participants familiar with AI had more positive perceptions, emphasizing the role of knowledge. Age affected professional impact but not preparedness. Gender, marital status, experience, and training showed no significant relationships with AI perceptions. These results underscore the need for tailored strategies to address specialty-specific needs and enhance AI literacy.

## Data Availability

The original contributions presented in the study are included in the article/supplementary material. Further inquiries can be directed to the corresponding author.
